# A viral genome wide association study and genotypic resistance testing in patients failing first line antiretroviral therapy in the first large countrywide Ethiopian HIV cohort

**DOI:** 10.1186/s12879-019-4196-8

**Published:** 2019-07-01

**Authors:** Nigus Fikrie Telele, Amare Worku Kalu, Solomon Gebre-Selassie, Daniel Fekade, Gaetano Marrone, Sebastian Grossmann, Ujjwal Neogi, Belete Tegbaru, Anders Sönnerborg

**Affiliations:** 1 Division of Clinical Microbiology, Department of Laboratory Medicine, Karolinska Institutet, Karolinska University Hospital, Alfred Nobels Allé 8, 141 83 Huddinge, Stockholm, Sweden; 2 Department of Microbiology, Immunology and Parasitology, Addis Ababa University, Addis Ababa, Ethiopia; 3 Department of Medicine, Addis Ababa University, Addis Ababa, Ethiopia; 4 Department of Public Health Sciences, Karolinska Institutet, Stockholm, Sweden; 5 Division of Infectious Diseases, Department of Medicine Huddinge, Karolinska Institutet, Karolinska University Hospital, Stockholm, Sweden; 6 Wellcome Trust Sanger Institute, Cancer, Ageing and Somatic Mutation Programme, Cambridge, UK; 7 Ethiopian Public Health Institute, Addis Ababa, Ethiopia

**Keywords:** HIV-1 drug resistance, Near-full length genome, Genome wide association, Antiretroviral therapy, Countrywide HIV cohort, Ethiopia

## Abstract

**Background:**

Antiretroviral therapy (ART) was rolled-out in Ethiopia in 2005, but there are no reports on outcome of ART and human immunodeficiency virus drug resistance (HIVDR) at national level. We described acquired drug resistance mutations in *pol* gene and performed a viral genome wide association study in virologic treatment failure patients who started first line ART during 2009–2011 in the first large countrywide HIV cohort in Ethiopia.

**Methods:**

The outcome of tenofovir (TDF)- and zidovudine (ZDV)-based ART was defined in 874 ART naïve patients using the on-treatment (OT) and intention-to-treat (ITT) analyses. Genotypic resistance testing was done in patients failing ART (> 1000 copies/ml) at month 6 and 12. Near full-length genome sequencing (NFLG) was used to assess amino acid changes in HIV-1 *gag*, *pol*, *vif*, *vpr*, *tat*, *vpu*, and *nef* genes between paired baseline and month 6 samples.

**Results:**

High failure rates were found in ITT analysis at month 6 and 12 (23.3%; 33.9% respectively). Major nucleoside and non-nucleoside reverse transcriptase (NRTI/NNRTI) drug resistance mutations were detected in most failure patients at month 6 (36/47; 77%) and month 12 (20/30; 67%). A high rate of K65R was identified only in TDF treated patients (35.7%; 50.0%, respectively). No significant difference was found in failure rate or extent of HIVDR between TDF- and ZDV- treated patients. All target regions of interest for HIVDR were described by NFLG in 16 patients tested before initiation of ART and at month 6.

**Conclusion:**

In this first Ethiopian national cohort, a high degree of HIVDR was seen among ART failure patients, independent on whether TDF- or ZDV was given. However, the major reason to ART failure was lost-to-follow-up rather than virologic failure. Our NFLG assay covered all relevant target genes for antiretrovirals and is an attractive alternative for HIVDR surveillance.

**Electronic supplementary material:**

The online version of this article (10.1186/s12879-019-4196-8) contains supplementary material, which is available to authorized users.

## Background

In Ethiopia, over 700,000 people are currently estimated to live with human immunodeficiency virus (HIV), corresponding to an adult prevalence of 1.15% [1]. Since 2005, antiretroviral therapy (ART) has been widely accessible through the WHO public health approach [2, 3]; the first-line regimen consists of fixed-dose combinations (FDC) of two nucleoside/nucleotide reverse transcriptase inhibitors (NRTI; zidovudine (ZDV) or tenofovir (TDF) plus lamivudine (3TC) or emtricitabine (FTC)), and a non-nucleoside RTI (NNRTI; efavirenz (EFV) or nevirapine (NVP) [4]. About 420,000 people living with HIV (PLHIV) were on ART by 2016 (nearly 60% coverage) [5]. However, the absence of viral load monitoring in Ethiopia and the high proportion of lost-to-follow-up (LTFU) [6, 7] are predicted to lead to a high rate of treatment failure and emergence of drug resistance, as seen in other sub-Saharan African countries (sSA) [8].

Only a few studies with relatively small number of patients from limited geographical regions in Ethiopia have reported ART failure rates, including acquired HIV drug resistance (HIVDR) [9–12]. Although ART has been rapidly scaled up throughout the country, to the best of our knowledge, there is no data at the national level. Hence, using a large nationwide HIV cohort, we assessed treatment failure, including acquired HIVDR by genotypic resistance testing and performed viral genome wide association studies by near-full length genome (NFLG) sequencing. In addition, we evaluated our NFLG assay for its capacity to amplify all HIVDR target regions of interest since it is an attractive alternative for HIV drug resistance mutation (DRM) surveillance.

## Methods

### Patients

A total of 874 ART naïve patients were enrolled into the Advanced Clinical Monitoring (ACM) of ART in Ethiopia from seven university hospitals during 2009–2011 as we described elsewhere [13, 14]. As per the national guidelines [4], patients were given fixed dose combinations (FDC): TDF + 3TC + EFV (*n* = 389), TDF + 3TC + NVP (*n* = 78), ZDV + 3TC + EFV (*n* = 104), ZDV + 3TC + NVP (*n* = 258), stavudine (d4T) + 3TC + EFV (*n* = 23), d4T + 3TC+ NVP (*n* = 21), and abacavir (ABC) + 3TC + EFV (*n* = 1). Altogether TDF was given to 467 (53.4%), ZDV to 362 (41.4%), d4T to 44 (5%) and ABC to one patient (Table 1).

**Table 1:** Patient characteristics at baseline and virological treatment failure at month 6 and 12

Characteristics	All patients *n* = 874	GRT^a^		NFLG^b^ (*n* = 16)
		Month 6 (*n* = 47)	Month 12 (*n* = 30)	
Gender: female/male	527/347 (60/40%)	23/24 (49/51%)	17/13 (57/43%)	5/11 (31/69%)
Age: years (median, IQR)	33 (11)	30 (13)	30 (9)	31 (13)
WHO clinical stage				
stage I	149 (17.0%)	4 (8.5%)	3 (10.0%)	1 (6.3%)
stage II	223 (25.5%)	13 (27.7%)	8 (26.7%)	5 (31.3%)
stage III	372 (42.6%)	19 (40.4%)	12 (40.0%)	6 (37.5%)
stage IV	130 (14.9%)	11 (23.4%)	7 (23.3%)	4 (25.0%)
NRTI				
Tenofovir	467 (53.4%)	28 (59.6%)	16 (53.3%)	12 (75.0%)
Zidovudine	362 (41.4%)	15 (31.9%)	10 (33.3%)	4 (25.0%)
Stavudine	44 (5.0%)	4 (8.5%)	4 (13.3%)	0 (0.0%)
NNRTI				
Nevirapine	380 (43.5%)	24 (51.1%)	16 (53.3%)	10 (62.5%)
Efavirenz	494 (56.5%)	23 (48.9%)	14 (46.7%)	6 (37.5%)
Laboratory values				
CD4+ T-cells/μl: mean; SD	143; 87	108; 80	113; 87	86; 76
HIV RNA log10 copies/ml: mean; SD	5.2; 0.8	5.4; 0.9	5.2; 1	5.8; 0.8

Number of patients with viral failure whose virus was sequenced with ^a^genotypic resistance testing (GRT) or ^b^near-full length sequencing (NFLG) at baseline, month 6 and/or 12; NRTI: nucleoside analogue reverse transcriptase inhibitors; NNRTI: non-nucleoside-analogue reverse transcriptase inhibitors. GRT was successful in 47 out of 51 tested patients and 30 out of 33 tested patients at month 6 and month 12, respectively

### Treatment outcome measurements

The treatment outcomes at month 6 and 12 were determined by on-treatment (OT) and intention-to-treat (ITT) analyses. Two categories of virological treatment failures were defined as i) > 150 copies/ml (limit of detection of the assay); ii) > 1000 copies/ml (as per WHO definition). For ITT, treatment failure was defined as failure to attain viral suppression (as described for OT) or lost-to-follow-up (LTFU) including confirmed death, moved from study sites or similar reasons.

### Clinical and laboratory tests

Clinical, routine laboratory and CD4 T-cells were analysed at the study sites [4]. Viral load was quantified by MT 2000 real time PCR (Abbott, USA) (detection limit 150 copies/ml).

### Population-based sanger sequencing (PBSS)

PBSS was attempted on patients with viral load ≥1000 copies/ml at month 6 in 51 subjects and month 12 in 33 patients [14]. In brief, HIV RNA was extracted from 140 μl plasma. The first-round of PCR was done using JA203F-C (forward) and JA206R-C (reverse) primer pair, followed by the second-round PCR, using JA204F-C (forward) and JA205R-C (reverse) primer pair [15]. The amplified fragments were purified and sequenced with JA204F-C and JA205R-C primers plus PR2R (5′-GGATTTTCAGGCCCAATTTTTG-3′) and RT07 (5′-AAGCCAGGAATGGATGGCCCA-3′). Sequences were analysed using the BioEdit software version 7.2.6.1 (http://www.mbio.ncsu.edu/bioedit/bioedit.html). Acquired DRM were identified by the Stanford HIVdb Program (hivdb.stanford.edu). Genotypic drug resistance defined as the presence of ≥1 major amino acid substitution included in the 2015 International Antiviral Society–USA (IAS) mutation list [16] and the Stanford algorithm was used to predict drug susceptibility. Drug classes considered were NRTI, NNRTI, and protease inhibitors (PI).

### Near-full length genomes (NFLG)

NFLG sequencing was performed on plasma from baseline and month 6 of 16 randomly selected patients among virologic failure patients with VL > 1000 copies/ml at month 6 of whom 12 were given TDF and four ZDV (Table 1), as described earlier [17, 18]. In brief, the NFLG (HXB2: 790 to 9554) was amplified in two primary fragments of 5.5 kb and 3.7 kb with an overlap of 400 bp and sequenced with up to 23 primers. CAP3 Sequence Assembly Program with default parameter was used to assemble the final NFLG [19]. The first NFLG HIV-1C_ET_ sequence (U46016) described by our group was used as a reference [20]. A multiple sequence alignment with our NFLG sequences was generated with the reference genome in AliView ver. 1.17.1 software [21] and analysed with an *in-house* Perl script that recognized the nucleotide changes from the reference sequence and created a corresponding number code as per HXB2 coordinates (790 to 9417). The resulting matrix was plotted using the TraMineR package [22] in R ver. 3.1.2 [23] to obtain a diversity plot. Maximum likelihood phylogenetic analysis was performed using Molecular Evolutionary Genetics Analysis version 7.0 (MEGA 7) software.

### Identification of mutations

Using AliView ver 1.17.1 and BioEdit ver 7.2.6.1 softwares, we aligned nucleotides and amino acids generated for each gene from the paired samples and described the specific amino acid mutations, which had appeared at month 6. The protein alignments were manually reviewed to identify changed residues. As European guideline recommended we have used the Geno2pheno tools at FPR 10% cut-off (Geno2Pheno_FPR10%_) for prediction of tropism throughout the analysis [24].

### Statistical analysis

Descriptive statistics (mean, median, standard deviation, and percentiles for numerical variables; frequencies and percentages for categorical variables) were used to summarize sociodemographic, clinical and virological parameters. Treatment outcomes were compared between patients with different NRTI regimens by Chi-square or Fisher’s exact test. The prevalence and type of DRM were compared between patients with TDF- or ZDV-based regimens by Chi-square or Fisher’s exact test. *P*-value < 0.05 was considered statistically significant. Data analysis was performed using STATA software 14 (Stata Corp. College Station, Texas, USA).

## Results

### Outcome of TDF- and ZDV-based ART

Among TDF treated patients, OT analysis identified virologic failure in 52/350 (15.0%) and 34/350 (9.7%) at month 6 using the 150 copies/ml and 1000 copies/ml cut-off values, respectively. ITT analysis identified treatment failure in 129/426 (30.2%) and 111/426 (26.0%) patients, respectively (Table 2). For ZDV, the figures were 34/288 (11.8%), 19/288 (6.6%) in the OT; 83/337 (24.6%), and 68/337 (20.2%) in the ITT analysis, respectively. For d4T treated patients, OT and ITT identified 4/38 (10.5%) and 9/43 (20.9%) virologic failure in both viral load cut-off values, respectively. There was no statistically significant difference in outcome across the NRTI regimens.

**Table 2:** Treatment outcome at month 6 and 12 in relation to the nucleoside analogue used

Time point	All: *n* (%)	Tenofovir	Zidovudine	d4T	*p*-value
Baseline^a^	874	467/874; 53.4%	362/874; 41.4%	44/874; 5.0%)	NA
Month 6^b^	743/874; 85.1%	391/467; 83.7%	313/362; 86.5%	39/44; 88.6%	0.591
*with VL; still on treatment*	676/743; 91.0%	350/391; 89.5%	288/313; 92.0%	38/39; 97.4%	0.225
*VL > 150*^*c*^	90/676; 13.3%	52/350; 15.0%	34/288; 11.8%	4/38; 10.5%	0.956
*VL > 1000*^*c*^	57/676; 8.4%	34/350; 9.7%	19/288; 6.6%	4/38; 10.5%	0.957
*Died or LTFU*	131/874; 15.0%	77/426; 18.1%	49/337; 14.5%	5/38; 13.2%	0.912
*Died or LTFU + VL > 150*^*c*^	221/807; 27.4%	129/426; 30.2%	83/337; 24.6%	9/43; 20.9%	0.731
*Died or LTFU + VL > 1000*^*c*^	188/807; 23.3%	111/426; 26.0%	68/337; 20.2%	9/43; 20.9%	0.773
Month 12^b^	690/874; 78.9%	364/467 77.9%	289/362; 79.8%	37/44; 84.1%	0.730
*with VL; still on treatment*	459/690; 66.5%	219/364; 60.2%	207/289; 71.6%	33/37; 89.2%	**0.006**
*VL > 150*^*#*^	61/459; 13.3%	30/219; 13.7%	27/207; 13.0%	4/33; 12.1%	1.000
*VL > 1000*^*c*^	34/459; 7.4%	19/219; 8.7%	13/207; 6.3%	2/33; 6.1%	0.989
*Died or LTFU*	184/874; 21.1%	103/322; 32.0%	73/280; 26.1%	7/40; 17.5%	0.654
*Died or LTFU + VL > 150*^*c*^	245/643; 38.1%	133/322; 41.3%	100/280; 35.7%	11/40; 27.5%	0.623
*Died or LTFU + VL > 1000*^*c*^	218/643; 33.9%	122/322; 37.9%	86/280; 30.7%	9/40; 22.5%	0.550

^a^one additional patient was given abacavir; ^b^still remaining in the study at month 6 or 12; ^*c*^Although 67 patients continued receiving their treatment (TDF: 41, ZDV: 25, and d4T:1) during the first 6 months follow up period, they were excluded from analyses due to lack of viral load data. Hence, in the ITT analysis 426, 337, and 43 TDF-, ZDV-, and d4T-treated patients were considered, respectively. Similarly, 231 patients were receiving their treatment (TDF: 145, ZDV: 82, and d4T:4) during the 12 months follow up period, but they were excluded from the analysis due to lack of viral load data. Hence, in the ITT analysis 322, 280, and 40 TDF-, ZDV-, and d4T-treated patients were considered, respectively

At month 12, for TDF patients OT analysis showed virologic failure in 30/219 (13.7%) and 19/219 (8.7%) patients using the 150 copies/ml and 1000 copies/ml cut-off values, respectively. In ITT analysis treatment failure was found in 133/322 (41.3%) and 122/322 (37.9%) patients, respectively (Table 2). For ZDV, the figures were 27/207 (13.0%), 13/207 (6.3%), 100/280 (35.7%), and 86/280 (30.7%), respectively. For d4T, the figures were 4/33 (12.1%), 2/33 (6.1%), 11/40 (27.5%), and 9/40 (22.5%), respectively. There was no statistically significant difference in treatment outcome across the NRTI regimens. No statistically significant difference was found in treatment outcome at month 12 as well when patients failing TDF were compared with those failing ZDV.

### Acquired DRM detected by PBSS at month 6 and 12

At month 6, a total of 47 sequences were obtained from 28/34 (82.4%), 15/19 (78.9%), and 4/4 (100%) of the TDF-, ZDV-, d4T-failing patient samples with VL > 1000 copies/ml, respectively (Table 3). One to seven (median: three) major NRTI and/or NNRTI DRM (but no major PI DRM) were found in 36/47 (76.6%) samples: NRTI+NNRTI: *n* = 24 (66.7%); only NRTI: n = 2 (5.6%); only NNRTI: *n* = 10 (27.8%) (detailed description of the DRM presented on Additional file 1: Table S1). Twenty-one of the 28 (75.0%) TDF failure patients had DRM (NNRTI+NRTI: 17; NNRTI only: 4). K65R was found in 10 (35.7%) subjects. Six patients had TAM (TAM-1: 1; TAM-2: 5; TAM-1 + TAM-2: 1). No significant difference was found in viral load at month 6 for patients failing TDF with or without K65R. In ZDV patients, 12/15 (80.0%) had DRM (NNRTI+NRTI: 5; NNRTI only: 6, NRTI only: 1). No K65R was found in this group.

**Table 3:** Reverse transcriptase inhibitors associated drug resistance mutations (DRM) in patients failing therapy at month 6 and/or 12 by the type of first-line regimen

DRM at VL > 1000 copies/ml	Tenofovir	Zidovudine	*p*-value
Month 6			
*> 1000 copies/ml, readable sequence*	28/34; 82.4%	15/19; 78.9%	0.755
**NRTI**	17/28; 60.7%	6/15; 40.0%	0.195
TAM	6/28; 21.4%	4/15; 26.7%	0.695
K65R	10/28; 35.7%	0/15; 0.0%	**0.008**
M184I/V	9/28; 32.1%	4/15; 26.7%	0.713
Others (TDF: A62V, K70E, V75I, Y115F, F116Y; ZDV: L74 V)	7/28; 25.0%	1/15; 6.7%	0.142
**NNRTI**	21/28; 75.0%	11/15; 73.3%	0.903
**NRTI + NNRTI**	17/28; 60.7%	5/15; 33.3%	0.087
**None**	7/28; 25.0%	3/15; 20.0%	0.711
Month 12			
*> 1000 copies/ml, readable sequence*	16/17; 94.1%	10/12; 83.3%	0.347
**NRTI**	12/16; 75.0%	3/10; 30.0%	**0.024**
TAM	5/16; 31.3%	2/10; 20.0%	0.528
K65R	8/16; 50.0%	0/10; 0.0%	**0.007**
M184I/V	7/16; 43.8%	1/10; 10.0%	0.069
Others (TDF: A62V, K70E, V75I, Y115F)	4/16; 25.0%	0/10; 0.0%	0.086
**NNRTI**	13/16; 81.3%	4/10; 40.0%	**0.031**
**NRTI + NNRTI**	12/16; 75.0%	2/10; 20.0%	**0.006**
**None**	3/16; 18.8%	5/10; 50.0%	0.094

At month 12, a total of 30 sequences were obtained from 16/17 (94.1%), 10/12 (83.3%) and 4/4 (100%) of the TDF-, ZDV-, d4T-failing patients, respectively. One to eight (median: four) major NRTI and/or NNRTI DRM were found in 20 (66.7%) samples: NRTI+NNRTI: *n* = 16 (80.0%); only NRTI: n = 1 (5.0%); only NNRTI: *n* = 3 (15.0%). No significant difference was found in viral load at month 6 or month 12 for patients failing TDF with or without DRM and for patients failing ZDV with or without DRM. Thirteen of 16 (81.3%) TDF failure patients had DRM (NNRTI+NRTI: 12; NNRTI only: 1). K65R was found in eight (50%) patients and TAM-2 in five patients. In ZDV patients, 5/10 (50%) had DRM (NNRTI+NRTI: 2; NNRTI only: 2; NRTI only: 1). With regard to the number of DRM in sequences harboring such mutations, no difference was found between patients failing TDF or ZDV at month 6. However, at month 12 sequences harboring mutations from patients failing TDF had more DRM as compared to those failing ZDV (*p* = 0.017), NNRTI DRM (*p* = 0.037), and NRTI DRM (*p* = 0.040).

### Amino acid changes identified by NFLG

**Fig. 1:**
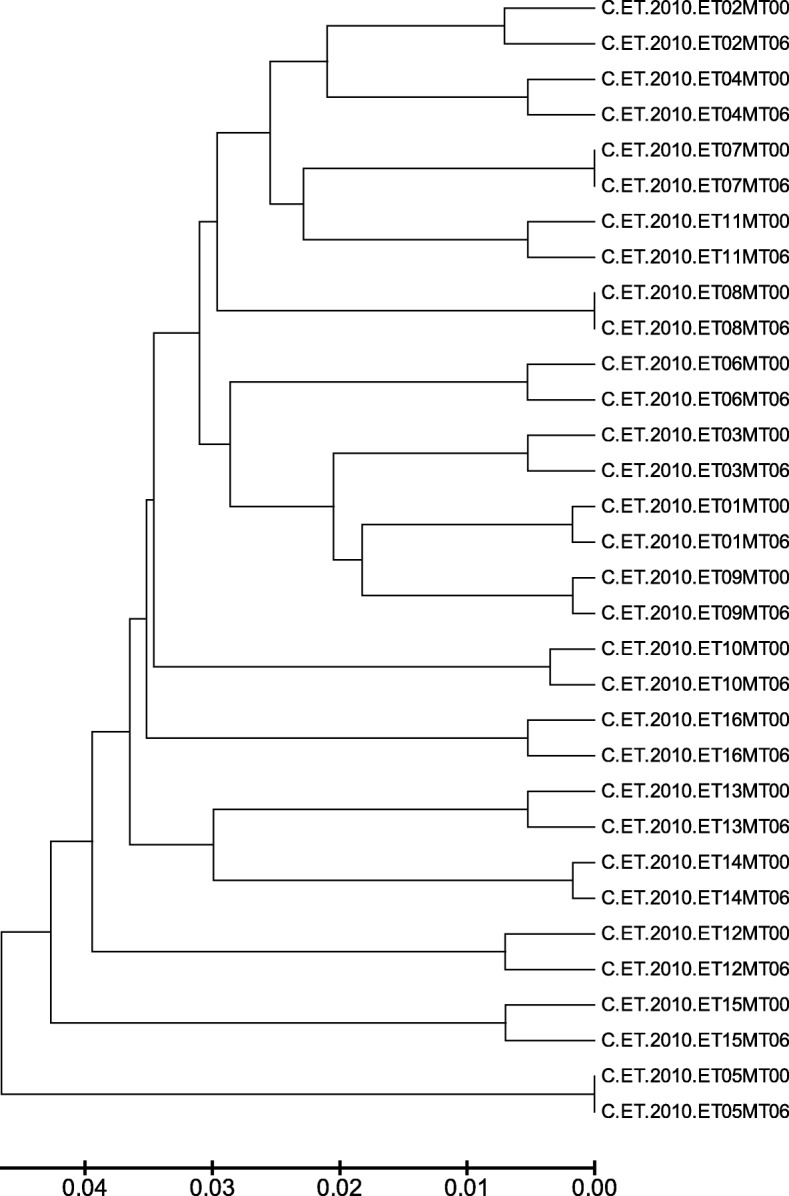
Maximum likelihood phylogenetic analysis of the baseline and month 6 NFLG sequences showing proper matching. A Neighbor-Joining tree was generated in MEGA with the Kimura 2-parameter method and full-length sequences of all successfully assembled samples. All final branches display a full bootstrap support of 100% confirming proper sample matching without cross-contamination and therefore all samples could be used for longitudinal analysis. The scale bar corresponds to 0.01 change per nucleotide

NFLG sequences including *gag*, *pol*, *vif*, *vpr*, *tat*, *vpu*, and *nef* were successfully generated in all 32 (16 paired) samples, except for the *nef* gene at month 6. Maximum likelihood phylogenetic analysis revealed proper matching of the paired NFLG sequences with 100% bootstrap support (Fig. 1). No sample had hypermutations and the analysis predicted only functional viruses. Analysis of the 16 baseline *pol* sequences showed no major DRM. No patient had PI- or integrase strand inhibitor (INSTI) DRM.

**Fig. 2:**
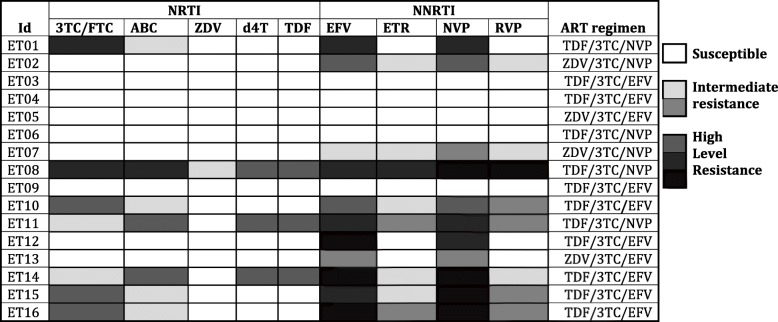
Predicted sensitivities to antiretroviral drugs at month 6

**Fig. 3:**
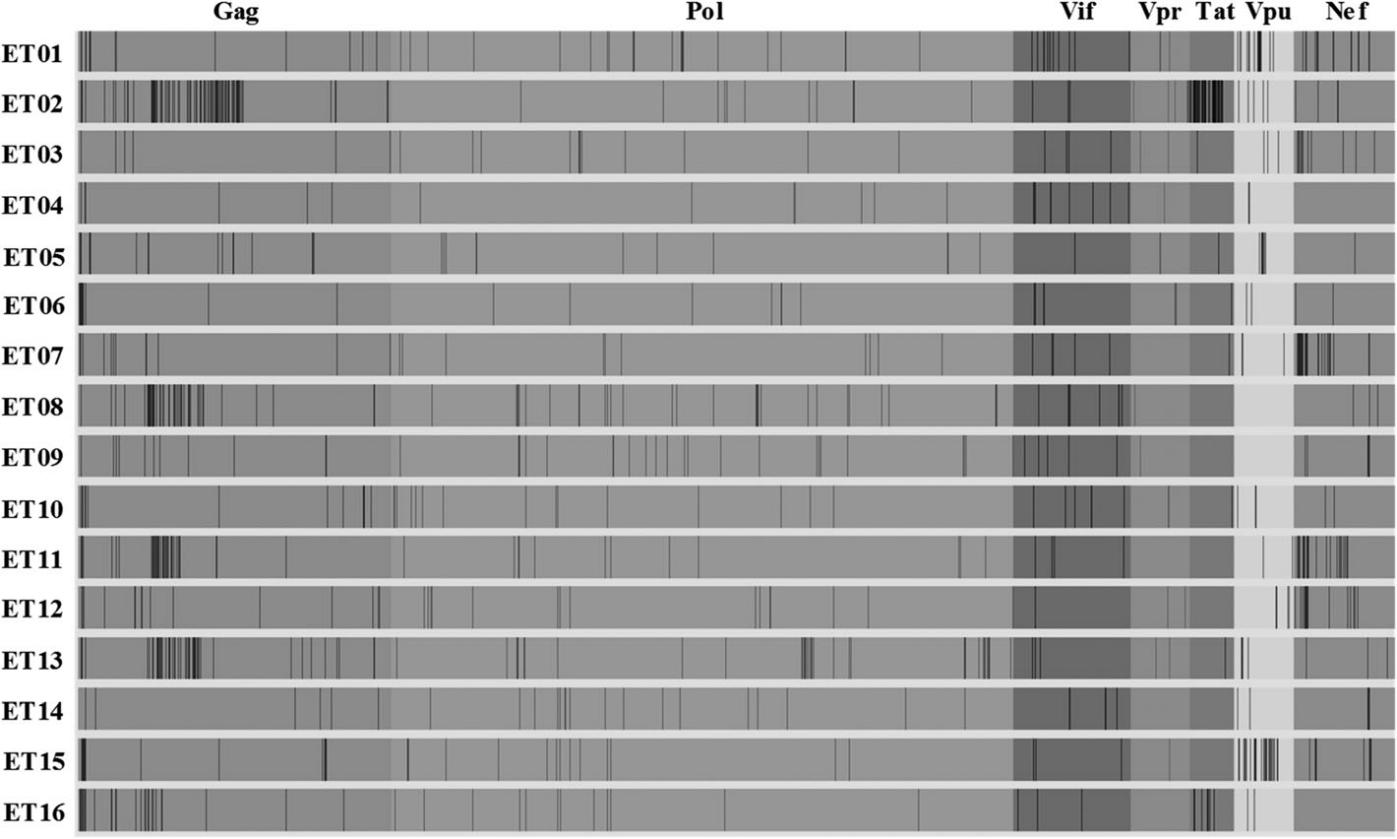
Amino acid mutations at month 6 in 16 paired samples by NFLG assay. The *nef* gene was analysed only in 15 patients (due to amplification failure for ET16 month 6 sample)

After six months of ART, eleven (68.8%) patients had acquired one to five (median: three) major DRM in *pol* (NRTI+NNRTI: 7; NNRTI: 4). The predicted sensitivities to NRTI and NNRTI are given on Fig. 2. Although none had known PI- or INSTI-associated DRMs, several other mutations were identified in the protease and integrase regions (A9P, E15K, A42E/T, K258 N, N278S/D, Y336C, G345A/S, K462R, and M532R/L) in two or three samples each. Four amino acid (GTIP/GALN/GTLV/GTLQ) insertions were displayed in the protease region at positions 48–55 (Fig. 3).

For *gag*, amino acid changes mostly clustered in the p6 and p17 regions. In the Gag-p6 region, A5G/E/P/P5S/A/R5A and K6S/P6A/S6K/E6K/V6E mutations were displayed in seven and five samples, respectively. PYKE tetra-peptide motif was found both at baseline and month 6 in nine (56.3%) of the 16 sample pairs on the C-terminal position of the p6 region. Duplicate tetra-peptide motif PTAP was identified in two samples at baseline, where the respective month 6 samples had single motif (deletion of one of the double motifs). A triple PTAP tetra-peptide motif was found in paired samples of a patient. The remaining 13 patients (81.3%) had only single P(T/S)AP motif in their paired samples. In the C-terminal of p17, 14 (87.5%) of the 16 patients displayed the R4S mutation. On the other hand, only one patient had a mutation (T375 M) in the p2/NC *gag* cleavage site.

Mutations were also identified in the *vif* (N36K/S *n* = 4; G101S/D *n* = 4, V31I *n* = 3, K33R, R34K, K63R, R92G, G101D *n* = 2) and in the *nef* gene (A15E/S *n* = 3, L20I/A/R *n* = 3; W5R/Q, K7N, C8S, V11P/G, R19A/P, D24A/G, K105 N/T, K118E/R *n* = 2). The t*at, vpu* and *vpr* showed very few amino acid changes (Fig. 3).

### Co-receptor tropism and long-terminal repeat (LTR)

The V3 loop was successfully sequenced in 13 baseline and 14 month 6 samples (eleven paired). The Geno2Pheno_FPR10%_ tool predicted 10/13 baseline viruses to be CCR5, two as CXCR4 tropic and one as CXCR4/CCR5 dual tropic. Of the 14 viruses at month 6, 12 were predicted as CCR5 and two as CXCR4 tropic. The CXCR4/CCR5 dual tropic virus at baseline switched to CXCR4 and the CXCR4 virus to CCR5 tropic.

Of the 16 baseline samples 15 (93.8%) displayed three nuclear factor kappa B (NF-kB) each in the enhancer region of the LTR of HIV-1C and the remaining one sample shows two NF-kB (which has short nucleotide sequence). Again 15 of the 16 (93.8%) month 6 samples displayed triple NF-kB, but one sample displayed large insertions in the LTR and showed four NF-kB instead.

## Discussion

In this first large countrywide study of ART outcome in Ethiopia, a high failure rate was identified in the ITT-analysis; around one-fourth at month 6 and one-third at month 12, whereas the OT-analysis revealed less than 10% of the participants failed virologically (> 1000 copies/ml) at month 6 and 12. Viral load is a gold standard for monitoring ART response and is a marker of the treatment outcome [25]. However, the optimal threshold for defining virologic failure and for switching ART regimens has not been well established in the setting of LMIC, and WHO recommended a threshold of 1000 copies/ml [26]. Below 1000 copies/ml, viral blips or intermittent low level viremia (50–1000 copies/ml) can occur during effective treatment, but their relevance in the LMIC setting has not been proven [27]. In our cohort, the baseline HIV RNA levels were high (mean 5.2, SD 0.8 Log10 copies/ml) although the levels were in line with what has been earlier reported from real-life cohorts in sub-Saharan Africa [28]. Thus, in view of a possible slower decay of viremia for very high viral load values the treatment failure rate due to viral rebound at month 6 may have been overestimated in our study. In addition, although some studies suggest that drug resistance present in patients with low level viremia could impact the long term treatment outcomes [29], others highlighted standard drug resistance testing may be unreliable and difficult to obtain among such patients [30].

This study confirms that early death and LTFU are major reasons to poor treatment outcome of ART in Ethiopia, as described from other sSA countries [31], although broad HIVDR to the first line regimens was common on those still on ART. In our study HIVDR was identified in 76.6 and 66.7% of virologic failure patient samples with VL > 1000 copies/ml at month 6 and 12, respectively as determined by PBSS. The treatment outcome of the different NRTI-based regimens did not differ, neither the extent of viral resistance at failure, although the K65R mutation was only found in TDF-treated patients. All relevant target regions for HIVDR were described in the subset of patients who were analysed by our NFLG.

In the WHO recommendations, TDF has since several years replaced thymidine analogues (ZDV and stavudine) in first-line regimens. As shown in our study, the introduction of TDF without virological monitoring may result in an extensive evolution of the K65R mutation, especially since it is preferentially selected by HIV-1C in ex vivo and in vitro analysis [32, 33]. Importantly, strains with the K65R may be transmitted further which may jeopardize future therapeutic and prophylactic use of TDF and of tenofovir alafenamide [34].

Because of its well-recognized toxicities and as per the WHO recommendation [3, 35], Ethiopia has amended its guidelines to initiate all new patients on non-d4T-based ART regiments. Accordingly, the use of d4T-based regimen has been observed reduced in our study into 5%. However, the retention rate observed among our patients who received this regimen was significantly higher than those who were on TDF-based and ZDV-based regimens (*p* < 0.05). In contrary to our observation, studies from resource-limited settings revealed that TDF-based regimens performed better than d4T, most notably with a significantly higher rate of LTFU for d4T patients [36].

Retention in care was low and undocumented mortality and self-transfer of patients are likely to have contributed. A possible way forward to improve treatment outcome could possibly be the use of long-acting drugs, such as the integrase inhibitor cabotegravir, which inhibits HIV-1C at least as efficient as HIV-1B [37], and the NNRTI rilpivirine. However, rilpivirine may not be an optimal drug in HIV-1C infected Ethiopians due to the high viral load in the majority of patients at diagnosis and the less binding efficacy to the HIV-1C reverse transcriptase [38]. Also, it has now become clear that the clearance of these two long-lasting drugs takes a very long time after cessation of therapy. Therefore the risk of development of resistance to both rilpivirine and cabotegravir is high if a patient is not adherent to the injection schedule [39].

Our HIV-NFLG sequencing assay was used to study the amino acid changes between paired samples from baseline and month 6 of virologic failure patients at several genes. It was found to efficiently amplify key HIV-1 drug target sites (PI, RTI, and IN) in all 32 tested samples, and the *env* and the LTR in the majority of patients. In addition, non-drug target sites like Gag and gp41 were sequenced which also can affect the drug efficiency [40, 41]. Thus, changes in the *gag* region may influence the efficacy of PI which is second line treatment option in Ethiopia. *Gag* mutations were found by NFLG in the majority of our patients at failure although the identified point mutations are not known to influence the response to PI. A PYKE tetra-peptide, which was found in the ALIX-binding motif of Gag-p6 at baseline in all patients, remained unaffected by the treatment in all subjects. In an earlier study, this tetra-peptide motif was observed among half of treatment naïve Ethiopian patients, but the status was not known among ART experienced patients [42]. In contrast, the PYKE motif was observed only in few sequences from South African and Indian ART naïve HIV-1C infected patients (1 and 3%, respectively). Therefore, it is important to elucidate the clinical relevance of the PYKE motif in terms of viral fitness and susceptibility to ART, especially to PI drugs with larger number of samples in HIV-1C subtypes.

Changes of the motif PTAP in Gag-p6 were also seen. Thus, a duplicate of the motif was found in baseline samples of two patients of which one motif was deleted from the virus of each patient at month 6. Also, a triple PTAP motif was detected in paired samples of one patient, which has not been described earlier. Subtype specific differences have earlier been observed in Gag-p6 with regard to the motif PTAP. In addition, a difference in ART outcome in relation to duplication of the PTAP motifs for HIV-1C has been reported [43]. The duplication probably restores the ALIX mediated virus release pathway, which is lacking in HIV-1C, as PTAP motif is thought to be a key player in viral budding [44]. After 6 months of ART, one of the duplicated motifs we initially observed in two baseline samples were deleted. This was not in line with a study that showed accumulations of long duplications within PTAP during ART in a high proportion of HIV-1C patients [43]. Therefore, a further study about the significance of this tetra-peptide motif on treatment outcome and its clinical relevance is recommended.

In the present study we have used our NFLG assay to analyse HIV drug resistance in an Ethiopian population and all relevant target regions for HIVDR were described. Although HIV resistance testing is presently not used clinically in most low- and middle income countries (LMIC), the method is an alternative for surveillance of HIV drug resistance. Earlier we have shown the negative impact of pretreatment drug resistance mutations on virologic outcome in our Ethiopian cohort [14]. Also, in view of the increased use of dolutegravir in the first line ART in LMIC and of boosted protease inhibitors in the second line ART it is important that the associated DRM can be surveilled.

It can also be noted that resistance to dolutegravir has been described outside the integrase, mainly in Nef and LTR overlapped region, in vitro and in patients failing monotherapy with dolutegravir [45]. In contrast to the findings in the *gag* gene, no common pattern of amino acid changes was seen in the other genes, with the exception of known RTI DRM in the *pol* gene. However, in all of the NFLG sequences we identified three NF-kB binding regions in the LTR which is unique for HIV-1C and described by us earlier [46]. Altogether the data suggest that our cost-effective NFLG assay has a potential for extended genotypic resistance testing, as compared to PBSS, and also for studies of the viral population dynamics in the HIV epidemic.

## Conclusions

In conclusion, although the retention in care was low in this first countrywide Ethiopian cohort no differences was found between patients given TDF- or ZDV-based regimens with regard to treatment outcome or level of drug resistance. A broad RT-inhibitor resistance was found in three quarters of the patients who were on-treatment with virologic failure. Our NLFG assay was shown to efficiently amplify genes of known or potential relevance for HIV drug resistance and is an attractive alternative for such surveillance in low and middle income countries.

## Additional file

Additional file 1: **Table S1.** Drug resistance mutations (DRM) associated with reverse transcriptase inhibitors in patients failing at month 6 and/or 12 by the population-based Sanger sequencing (PBSS) assay. (DOCX 18 kb)

## Data Availability

All data generated during this study are included in this published article and its supplementary information file. The *pol* sequences generated and analysed during the current study are available in the GenBank (accession numbers: MH666276 – MH666351).

## Abbreviations

3TC: Lamivudine
ACM: Advanced Clinical Monitoring
ART: Antiretroviral therapy
DRM: Drug resistance mutations
EFV: Efavirenz
FDC: Fixed-dose combinations
FTC: Emtricitabine
GRT: Genotypic resistance testing
HIV: Human immunodeficiency virus
HIV-1C: HIV-1 subtype C
HIVDR: Human immunodeficiency virus drug resistance
INSTI: Integrase strand inhibitors
ITT: Intention-to-treat
LTFU: Lost-to-follow-up
LTR: Long-terminal repeat
NFLG: Near-full-length genome sequencing
NNRTI: Non-nucleoside reverse transcriptase
NRTI: Nucleoside reverse transcriptase
NVP: Nevirapine
OT: On-treatment
PBSS: Population-based Sanger sequencing
PI: Protease inhibitors
TDF: Tenofovir
VL: Viral load
