# Supplementary material for: A viral genome wide association study and genotypic resistance testing in patients failing first line antiretroviral therapy in the first large countrywide Ethiopian HIV cohort
Source: BMC Infect Dis. 2019 Jul 1;19:569. doi: 10.1186/s12879-019-4196-8 (PMC6604127; doi:10.1186/s12879-019-4196-8)
Supplement: Supplementary file 1 — Table S1. Drug resistance mutations (DRM) associated with reverse transcriptase inhibitors in patients failing at month 6 and/or 12 by the population-based Sanger sequencing (PBSS) assay. (DOCX 18 kb) [file 12879_2019_4196_MOESM1_ESM.docx]

**Additional file 1: Table S1**. Drug resistance mutations (DRM) associated with reverse transcriptase inhibitors in patients failing at month 6 and/or 12 by the population-based Sanger sequencing (PBSS) assay.

| **Month 6** | | | **Month 12** | | |
| --- | --- | --- | --- | --- | --- |
| **SeqID** | **NRTI-DRM** | **NNRTI-DRM** | **SeqID** | **NRTI-DRM** | **NNRTI-DRM** |
| ET01P | None | K103N | NA | LTFU | LTFU |
| ET02P | M184V | V106M,G190A | ET02P | M184V | V106M, G190A |
| ET04P | None | G190S | ET04P | None | None |
| ET06P | K65N,K70R,V75I,F116Y,K219Q | E138A | ET06P | None | None |
| ET09P | K219KQ | K103N | ET09P | A62V, K65R, K219Q | K103N,V106M,Y181C |
| ET10P | A62V,K65R,M184V | K103N,Y181C | NA | Dead | Dead |
| ET11P | K65R, Y115F, K219N | G190E | ET11P | A62V, K65R, V75I, Y115F, K219N | G190E |
| ET12P | K65R | V106M, V179E, Y188C | ET12P | K65R | V106M, V179E, Y188C |
| ET16P | None | None | ET16P | A62AV,K65R,M184V | Y181C,G190A |
| ET18P | None | G190A | ET18P | None | K103KN,G190A |
| ET19P | M184V | K103N, V108I, P225H | ET19P | M184V, T215ALPV, K219KQ | K103N, K238T |
| ET20P | M184I,L210W | K103N,E138G,Y181C,G190A | NA | No VL | No VL |
| ET21P | K219Q | None | NA | No VL | No VL |
| ET23P | M184I | L100I, K103N | ET23P | K65R, M184I | L100I, K103N, M230L |
| ET24P | K65R | Y181C, G190GA, K238T | ET24P | D67DG, K70E, M184V | A98AG, Y181C, G190A, H221HY,K238KT |
| ET25P | K65R | V106M,V179D,Y181C | NA | LTFU | LTFU |
| ET26P | M184V | K103N,V108I,P225H | NA | VL<150 copies/ml | VL<150 copies/ml |
| ET27P | M184V | V108I,Y181C | NA | VL<1000 copies/ml | VL<1000 copies/ml |
| ET28P | None | K103N,V106M | NA | No VL | No VL |
| ET29P | None | K103N,V106M | NA | No VL | No VL |
| ET30P | K219KQ | K103N | NA | Dead | Dead |
| ET31P | L210LW | P236PL | NA | VL<1000 copies/ml | VL<1000 copies/ml |
| ET32P | K219Q | None | NA | VL<150 copies/ml | VL<150 copies/ml |
| ET33P | None | K103N | NA | LTFU | LTFU |
| ET34P | None | K101KE,K103KN | NA | No VL | No VL |
| ET35P | K65R,M184I | Y181C,M230L | NA | LTFU | LTFU |
| ET36P | None | K101KE | NA | VL<1000 copies/ml | VL<1000 copies/ml |
| ET37P | K65R | Y181C,G190A | ET37P | K65R,M184MV | K101E,Y181C,G190A |
| ET38P | Y115YF,M184V,T215S,K219KE | Y181C,H221Y | ET38P | K65R,Y115F,M184V,T215S | Y181C,H221Y |
| ET39P | None | K103N | NA | VL<1000 copies/ml | VL<1000 copies/ml |
| ET41P | K65R,Y115F | K103N,V106M,Y181C | NA | Dead | Dead |
| ET42P | K70KR,M184V | A98G,Y181C,G190A | NA | LTFU | LTFU |
| ET43P | V75I,M184MI | V106M,V179VD,Y188YC | ET43P | D67G,K70E | V106M,V179D,Y181C |
| ET44P | M184V | K101KE,V106M,G190A | NA | Dead | Dead |
| ET45P | None | K101KE | ET45P | T215TS,K219Q | Y188YN |
| ET46P | K65R,K70KE,M184IV | K103N,Y181C,G190S,F227L | NA | No sample | No sample |
| ET55P | L74V,M184V,K219N | K101KE,G190E | NA | No sample | No sample |
| NA | Amplification failure | Amplification failure | ET05P | None | V106M |
| NA | VL<150 copies/ml | VL<150 copies/ml | ET49P | None | K103KN,V179T,G190GA |
| NA | VL<150 copies/ml | VL<150 copies/ml | ET50P | M184V | K103N,H221Y |
| NA | VL<150 copies/ml | VL<150 copies/ml | ET52P | M184I | K101E,Y181C |
| NA | VL<150 copies/ml | VL<150 copies/ml | ET53P | K219Q | E138A |
| NA | VL<150 copies/ml | VL<150 copies/ml | ET56P | L210LW | None |
| NA | VL<1000 copies/ml | VL<1000 copies/ml | ET213P | K65R | V106M,V179D |
